# Clarifying the controversial risk-benefit profile of soluble ACE2 in COVID-19

**DOI:** 10.1186/s13054-020-03097-w

**Published:** 2020-07-06

**Authors:** Melvin Khee Shing Leow

**Affiliations:** 1 Lee Kong Chian School of Medicine, Nanyang Technological University, Singapore, Republic of Singapore; 2 Duke-NUS Medical School, Singapore, Republic of Singapore; 3 Department of Endocrinology, Division of Medicine, Tan Tock Seng Hospital, Singapore, 308433 Republic of Singapore

To the Editor:

In the timely report on age and gender differences in soluble angiotensin-converting enzyme 2 (sACE2) among COVID-19 patients, Sward et al. [1] elegantly showed that adults and men exhibited higher plasma concentrations of sACE2 relative to children and women, respectively. They assumed that high sACE2 levels reflect high membrane-bound ACE2 (mACE2) and/or ADAM-17 expression levels, thereby connoting a high susceptibility for COVID-19 infection. Yet, this has not been proven conclusively. First, mACE2 rather than sACE2 functions as the key portal of entry for SARS-CoV-2. Unlike plasma sACE2 which lacks critical membrane machinery for cell invasion, mACE2 homodimers dock with S-glycoprotein trimers of SARS-CoV-2 and cleave S into S1 (which binds ACE2 peptidase domain) and S2 (responsible for membrane fusion) for subsequent receptor-mediated endocytosis of virion particles by membrane-bound serine protease TMPRSS2 and lysosomal L-cathepsin [2]. sACE2 probably plays no role in orchestrating the cellular entry of SARS-CoV-2 without the molecular cooperativity of TMPRSS2 and L-cathepsin. Secondly, plasma ACE2 arises from shedding of mACE2 via ADAM-17 protease induced by angiotensin-II acting via AT-1 receptors [3]. Blockade of AT-1 receptors by angiotensin-receptor blockers (ARBs) inhibits this process and leads to higher mACE2 expression and lower sACE2, a situation that fueled initial concerns over ARBs possibly accentuating COVID-19 risks [4]. Hence, increased shedding of ACE2 should expectedly lead to lower mACE2 and correspondingly higher sACE2 and vice versa; this implies an inverse relationship between sACE2 and mACE2 rather than a direct correlation as posited by Sward et al. [1].

Finally, the authors merely portrayed the perceived negative connotation and prognosis of elevated sACE2 levels while omitting a critical appraisal of how its blood levels may paradoxically correlate with potential benefits, given that sACE2 is not only unlikely to influence viral invasion per se, but it can function as decoy ligands to sequester SARS-CoV-2 away from mACE2 which internalize docked viruses via membrane-associated enzyme dynamics that determines SARS-CoV-2 tissue tropism. Additionally, sACE2 levels can cleave circulating angiotensin-II to Ang-(1-7) and increase the systemic protective effects of ACE2/Ang-(1-7)/Mas axis signaling to reduce disease morbidity. In this connection, recombinant human rhACE2 may thus be safely and therapeutically delivered intravenously as recently planned in a clinical trial of rhACE2 for a cohort of COVID-19 patients to curb coronavirus invasion [5]. Given such mechanistic considerations, the jury is still out to determine unequivocally if plasma sACE2 level is a reliable biomarker portending a predilection for COVID-19 or fatality from the disease.

## Authors’ response

Per Swärd^1^, Andreas Edsfeldt^2^, Anton Reepalu^3^, Lars Jehpsson^1^, Björn E. Rosengren^1^, and Magnus K. Karlsson^1^^1^Clinical and Molecular Osteoporosis Research Unit, Departments of Orthopedics and Clinical Sciences, Skåne University Hospital, Lund University, Malmö, Sweden ^2^Department of Cardiovascular Research-Translational Studies and Cardiology, Skåne University Hospital, Lund University, Sweden ^3^Department of Translational Medicine and Clinical Infection Medicine, Skåne University Hospital, Lund University, Malmö, Sweden

We welcome the insightful comments by Leow on our recent publication [1]. We agree with the comments that nothing can be proven in respect of COVID-19 based on our study, which was conducted on a population based COVID-19 free population, longitudinally followed from childhood until young adulthood [1]. In the first sentence, Leow refers to our cohort as a COVID-19 population which is not the case. As we inferred in our original publication, the inferences towards COVID-19 in our publication are only hypothesis-generating. We agree that studies examining sACE2 as a risk marker (to be preferred over risk factor) for severe COVID-19 are lacking and suggested that such studies should be undertaken. These or other studies should also investigate how mACE2 levels and ADAM-17 induced mACE2 shedding are related to sACE2 so that we may understand if and how this is related to why severe COVID-19 rarely affects children, and men to a greater extent than women [6]. Future studies should, as suggested by Leow in his comments, also include if higher physiological circulating sACE2 can protect against SARS-CoV-2 infection and severe COVID-19.

## Data Availability

Not applicable.
